# Tension pyopneumothorax caused by *Parvimonas micra*: a case report

**DOI:** 10.1186/s13019-023-02239-9

**Published:** 2023-04-10

**Authors:** Yoshihito Iijima, Shun Iwai, Nozomu Motono, Hidetaka Uramoto

**Affiliations:** Department of Thoracic Surgery, Kanazawa Medical University, 1-1 Daigaku, Uchinada-Machi, Kahoku-gun, Ishikawa 920-0293 Japan

**Keywords:** Tension pyopneumothorax, *Parvimonas micra*, Decortication, Surgery, Pulse lavage irrigation system

## Abstract

Tension pyopneumothorax is a rare and life-threatening complication of pneumonia, lung abscess, and empyema, and immediate thoracic drainage or surgery is required. A 70-year-old man presented to another hospital 2 weeks after exacerbation of dyspnea and anorexia. Chest X-ray imaging revealed leftward deviation of the mediastinum, pleural effusion, and collapse of the right lung. The patient was referred to our hospital for surgical treatment. He underwent chest drainage immediately after the transfer. The patient’s blood pressure was elevated after drainage. Chest X-ray imaging showed improvement in the mediastinal deviation, but expansion failure of the lung occurred. Debridement and parietal and visceral decortications were performed under thoracotomy. The thoracic cavity was irrigated using a pulse lavage irrigation system with 12,000 mL of saline. The patient underwent fibrinolytic therapy with intrathoracic urokinase postoperatively because of persistent high inflammatory marker levels and multilocular pleural effusion. *Parvimonas micra* was detected in the preoperative pleural fluid culture. He was discharged on postoperative day 22 and followed up as an outpatient afterwards. Two years have passed since the surgery, and there has been no recurrence of empyema. Decortication of the parietal and visceral pleura and irrigation using a pulse lavage irrigation system were effective.

## Background

Tension pyopneumothorax (TPPTx) is a rare and life-threatening complication of pneumonia, lung abscess and empyema [1]. Empyema occurs when there are purulent exudates within the pleural space, which may or may not be associated with air or gas. TPPTx occurs when an empyema causes pneumothorax. Large amounts of purulent exudates and gas can cause lung collapse and shift the mediastinal organs, such as the heart, lungs, and trachea. Increased intrathoracic pressure can reduce the venous return with secondarily decreased cardiac output, and mediastinal deviation can compress the contralateral lung, leading to an emergent situation [2, 3]. Therefore, immediate thoracic drainage or surgery is often required. Herein, we report a successful surgical treatment of TPPTx caused by *Parvimonas micra*.

## Case presentation

**Fig. 1:**
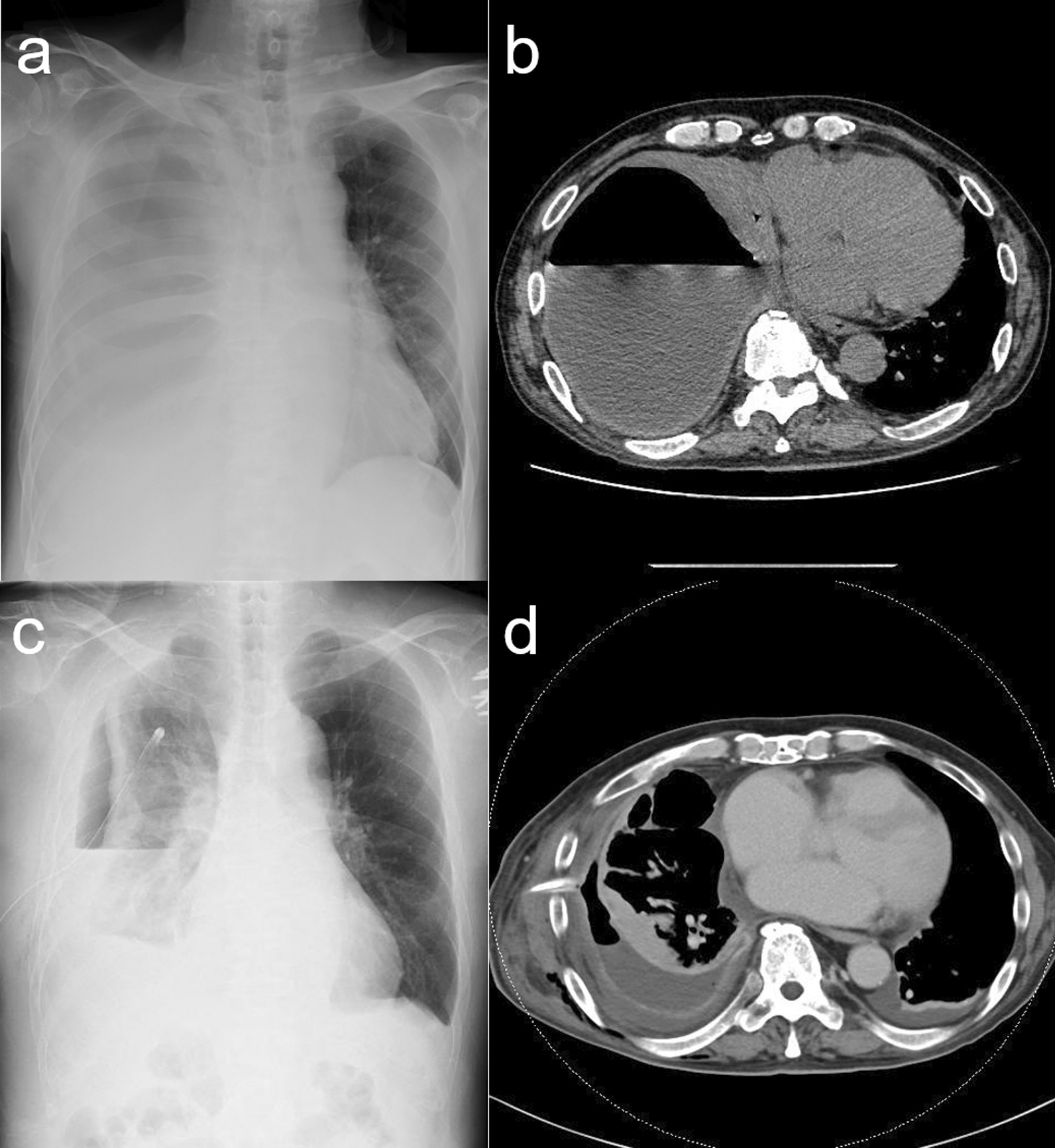
**a** Chest X-ray imaging and **b** computed tomography (CT) at the first visit. Chest X-ray imaging revealed right lung collapse and pleural effusion, and leftward deviation of the mediastinum. (Supine, anterior–posterior view) Chest CT revealed pleural effusion with air-fluid level and collapse of the right lung. **c** Chest X-ray and **d** CT imaging after drainage showed improvement in the mediastinal deviation and an expansion failure of the lung

**Fig. 2:**
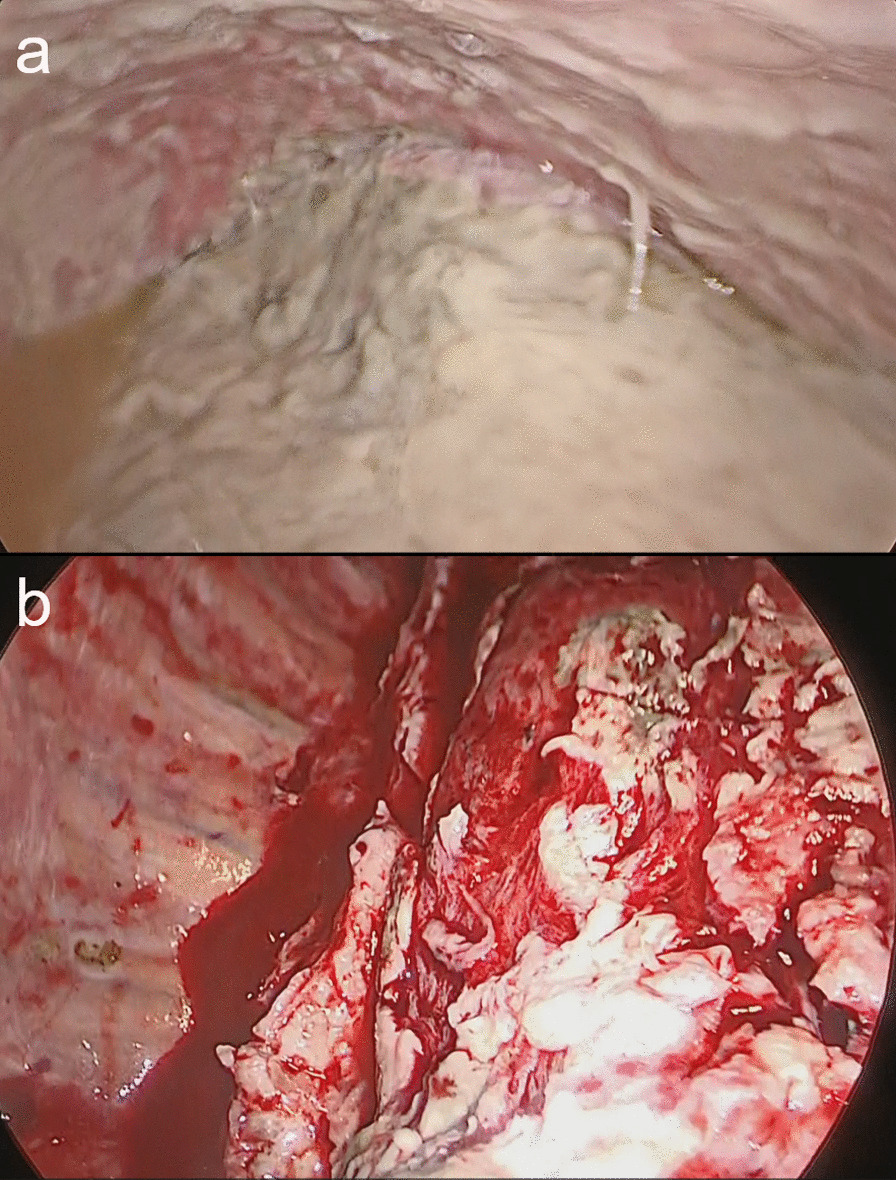
Intraoperative images. **a** The parietal and visceral pleura were markedly thickened. There was no fistula or air leaking from the parenchyma at the beginning of the surgery. **b** After parietal and visceral decortication. The parenchyma itself was normal without inflammation or abscess

**Fig. 3:**
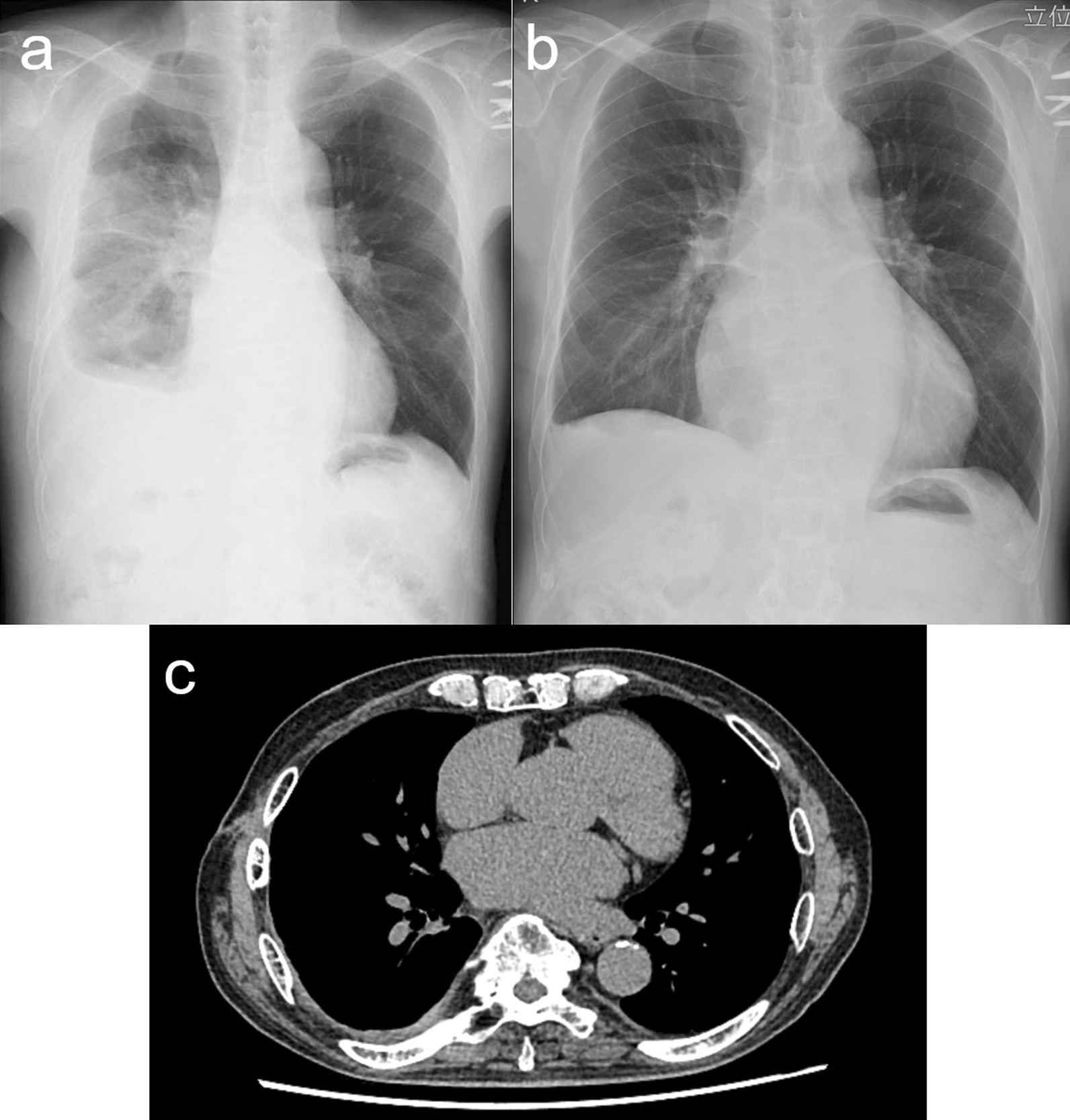
Postoperative images. **a** Chest X-ray at the discharge. **b** Chest X-ray and **c** computed tomography images at the 2-years follow-up examination

A 70-year-old man presented to the hospital 2 weeks after experiencing exacerbation of dyspnea and anorexia. His medical history included comorbidities, such as hypertension, atrial fibrillation, untreated dental caries, and periodontitis. He had a body temperature of 37.6 °C, blood pressure of 92/58 mmHg, and heart rate of 105 bpm. A chest X-ray (anterior–posterior view) revealed right lung collapse and decreased radiolucency of the right thoracic cavity with leftward deviation of the mediastinum (Fig. 1a). Chest computed tomography revealed pleural effusion with air-fluid level and collapse of the right lung (Fig. 1b). No mediastinal air was observed. White blood cell count and C-reactive protein levels increased markedly to 21,900 /MCL and 24.03 mg/dL, respectively. Procalcitonin level was elevated to 1.90 ng/mL. Thoracentesis was performed and 860 mL of the pleural fluid was aspirated. The pH of the pleural effusion decreased markedly down to 7.1. After initiating meropenem (MPEM) administration (3.0 g/day), the patient was referred to our hospital for surgical treatment. He underwent chest drainage immediately after the transfer. From the tube, 2000 mL of foul-smelling pus drained without air leakage. The patient’s blood pressure increased to 124/74 mmHg. Chest X-ray imaging showed improvement in the mediastinal deviation; however, an expansion failure of the lung was noted, and surgery was planned (Fig. 1c, d). There was no fistula or air leaking from the parenchyma at the beginning of the surgery (Fig. 2a). We first attempted thoracoscopic debridement. However, the parietal and visceral pleura were markedly thickened. Therefore, we converted to thoracotomy and performed parietal and visceral decortication. The parenchyma itself was normal without inflammation or abscess (Fig. 2b). Thoracic cavity was irrigated using a pulse lavage irrigation system with 12,000 mL of saline. An apical and a basal drain were placed over the lung apex and diaphragm. The operation time was 161 min, and the volume of blood loss was 600 mL. The apical drain was removed on postoperative day (POD) 3. On PODs 5 and 13, the patient underwent fibrinolytic therapy using intrathoracic urokinase administration to promote lung expansion because of persistent high inflammatory marker levels and multilocular pleural effusion. *P. micra* was detected in the preoperative pleural fluid culture. Based on the pleural effusion culture, it was determined to be the causative bacterium, and antibiotics were de-escalated from MPEM to metronidazole (1500 mg/day) on POD 8. The basal drain was removed on POD 20, because no bacteria were detected in either of the two pleural fluid cultures. The patient was discharged on POD 22 (Fig. 3a). The patient is under careful follow-up as an outpatient; at present, 2 years have passed since the operation, and there has been no recurrence of empyema (Fig. 3b, c).

## Discussion

TPPTx is a rare and life-threatening condition that was previously reported only in 20 cases, including our case [1, 3–20] (Table 1). Pyothorax has a high mortality rate (15%), and 30% of cases require early and appropriate treatment, such as surgical drainage of the pleural space [21]. Of the 20 previously-reported TPPTx cases of (five women and 15men), nine (45%) had a serious course, including cardiopulmonary arrest, shock, respiratory failure, and sepsis, and two of them had a fatal outcome. Of the 17 patients with a confirmed prognosis, three (17.6%) died. The average age was 52.1 (standard deviation: 17.6) years. Four cases involved the gastrointestinal tract (gastropleural fistula, esophageal rupture), four cases had lung infection (lung gangrene, echinococcal cyst, pulmonary nocardiosis, and aspiration pneumonia), four cases underwent treatment for malignancies, and three cases had a history of immunocompromising conditions (human immunodeficiency viral infection, steroid addict). Facultative or obligatory anaerobes were identified from pleural effusion cultures in 10 of 15 previously-described cases.

**Table 1:** The previous case reports of tension pyothrax and pyopneumothorax in adult

Case		Age		Sex		Background		Etiology		Culture of PE	Outcome						References	
											Fatal complication		Treatment		Prognosis			
1		20		F		Pharingitis		Unknown: air way tear or pharyngeal-mediastinal-pleural comunication?		*Streptococcus pyogenes*	–		Antibiotics, drainage		Alive		[4]	
2		28		F		HIV positive		ND		ND	CPA		Resuscitation, drainage		ND		[3]	
3		47		F		RTx for breast cancer		Gastropleural fistula		ND	CPA		Resuscitation		Alive		[5]	
4		65		F		MALT lymphoma		Gastropleural fistula		*Gram positive bacilli*	–		Drainage, surgery; total gastrectomy		Dead		[6]	
5		65		F		DM		–		*Actinomyces sp, mixed anaerobes*	–		Antibiotics, drainage, intrathoracic urokinase		Alive		[7]	
6		30		M		Hodgkin's disease		Left lung gangrene		ND	Septic shock		Surgery; fenestration		Alive		[8]	
7		31		M		Drug abuse		Unknown: pneumonia?		*mixed anaerobes*	–		Antibiotics, drainage		Alive		[9]	
8		35		M		–		–		*Staphylococcus sp.*	–		Antibiotics		Alive		[10]	
9		38		M		Pulmonary echinococcosis		Ruptured echinococcal cyst		*Acinetobacter baumanii* (sptum)	–		Antibiotics, drainage		Alive		[11]	
10		42		M		21 trisomy, DM		ND		failed to identify	CPA		Resuscitation, drainage		Dead		[1]	
11		45		M		Unknown		ND		*prevotella denticola*	Shock, RF		Antibiotics, drainageintubation and mechanical ventiration		ND		[12]	
12		49		M		ND		–		*Streptococcus viridans, Peptococcus*	–		Antibiotics, drainage		ND		[13]	
13		59		M		Aspiration pneumonia, Parkinson's disease		Bronchopleural fistula		*Prevotella and Wolinella sp.*	Shock, RF		Surgery; drainage and decortication		Alive		[14]	
14		62		M		Cerebral infarction sequelae		ND		*Streptococcus viridans, Morganella morganii*	Shock?, RF?		Antibiotics, drainage, intubation and mechanical ventiration		Alive		[15]	
15		68		M		DM, Alcoholic hepatitis, IP treated with a steroid		–		ND	Septic shock		Drainage, ND other treatment		Alive		[16]	
16		68		M		Total gastrectomy for gastric cancer, CTx for esophageal cancer		Ruptured lung abscess		*Klebsiella oxytoca, α-Streptococcus*	Shock, RF		Surgery; left lower lobectomy, decortication		Alive		[17]	
17		69		M		–		Esophageal diverticulum rupture		*Candida albicans, Microaerphilic streptococci, Escherichia coli*	Sepsis		Antibiotics, drainagesurgery; feeding jejunostomy, debridement, pharyngostomy		Dead		[18]	
18		74		M		Pulmonary nocardiosis, steroid therapy for autoimmune hepatitis		Ruptured lung abscess		*Nocardia pseudobrasiliensis*	–		Drainage, surgery; left lower lobectomy		Alive		[19]	
19		77		M		Barrett's esophagus		Esophagopleural fistula		ND	–		Drainage, surgery; distal esophagectomy		Alive		[20]	
20		70		M		HT, Af, dental caries, periodontitis		–		*Parvimonous micra*	–		Surgery; debridement, decortication		Alive		Our case	

*PE* pleural effusion, *F* female, *M* male, *HIV* human immunodeficiency virus, *RTx* radiotherapy, *MALT* mucosa-associated lymphoid tissue, *DM* diabetes mellitus, *ND* not described, *IP* interstitial pneumonia, *CTx* chemotherapy, *HT* hypertension, *Af* atrial fibrillation, *PTx* pyothorax, *PPTx* pyopneumothorax, *CPA* cardiopulmonary arrest, *RF* respiratory failure

*Parvimonas micra* is a bacterial flora in the oral cavity and gastrointestinal tract, and can be a pathogenic bacterium for chronic periodontal disease, alveolar pyorrhea, peritonsillar abscess, chronic sinusitis, chronic otitis media, and pulmonary suppuration [24]. Cobo et al. [24] reported pleurisy in three (9.6%) out of 31 cases of *P. micra* infection, and intrathoracic infection was relatively rare. *Parvimonas micra* is part of the oral flora, and 16 out of the 31 aforementioned cases had comorbidities, such as periodontitis and dental caries, dental procedures, or tooth extraction [24]. This patient had untreated dental caries and periodontitis, which were suspected to be related to TPPTx occurrence.

Empyema surgery is based on debridement and lavage of the pleural cavity. Recently, the effectiveness of using pulse lavage irrigation system in the treatment of empyema has been reported [22, 23]. Pulse irrigation uses high water pressure and can wash the area of its application without damaging the surrounding soft tissues, such as nerves and blood vessels. Moreover, the fibrin and necrotic tissues can be easily removed and washed. It has been reported that 90% of patients with acute empyema in the fibrinopurulent phase were cured completely without recurrence after pulse irrigation [22]. This case was considered to be in the late fibrinopurulent phase to the chronic organizing phase. It was observed that decortication and pulse irrigation were effective in cleaning the thoracic cavity.

In conclusion, we successfully treated a rare case of TPPTx caused by *P. micra* infection*.* The patient developed TPPTx owing to gas production from an anaerobic bacterial infection. Decortication of the parietal and visceral pleura and irrigation using a pulse lavage irrigation system seemed to be efficient in the treatment of the condition.

## Data Availability

The data underlying this article are available in the article.

## Abbreviations

TPPTx: Tension pyopneumothorax
MEPM: Meropenem
POD: Postoperative day
*P. micra*: *Parvimonas micra*
