# Correction: Tension pyopneumothorax caused by parvimonas micra: a case report

**DOI:** 10.1186/s13019-023-02291-5

**Published:** 2023-05-17

**Authors:** Yoshihito Iijima, Shun Iwai, Nozomu Motono, Hidetaka Uramoto

**Affiliations:** Department of Thoracic Surgery, Kanazawa Medical University, 1-1 Daigaku, Uchinada-Machi, Kahoku-gun, Ishikawa, 920-0293 Japan

**Correction to: Journal of Cardiothoracic Surgery (2023) 18:1**

10.1186/s13019-023-02239-9

Following publication of the original article [1], In this article the title was incorrectly given as ‘13019Tension pyopneumothorax caused by Parvimonas micra: a case report’ but should have been ‘Tension pyopneumothorax caused by Parvimonas micra: a case report’.

The original article has been corrected.
